# Expansion of the known distribution of the coastal tailed frog, *Ascaphus truei*, in British Columbia, Canada, using robust eDNA detection methods

**DOI:** 10.1371/journal.pone.0213849

**Published:** 2019-03-14

**Authors:** Jared Hobbs, Jessica M. Round, Michael J. Allison, Caren C. Helbing

**Affiliations:** 1 Hemmera Envirochem Inc., Victoria, British Columbia, Canada; 2 Department of Biochemistry and Microbiology, University of Victoria, Victoria, British Columbia, Canada; Leibniz-Institute of Freshwater Ecology and Inland Fisheries, GERMANY

## Abstract

The coastal tailed frog (*Ascaphus truei*) is endemic to the Pacific Northwest of North America and is listed as a species of Special Concern under the Canadian *Species at Risk Act*. Its range is limited to British Columbia where it occurs widely west of the Coast Mountain Ranges extending north almost to the Alaskan Panhandle. The present study focused on surveying within the Cayoosh, Bridge (Shulaps), Seton, Anderson, Carpenter, and Downton Lake drainages. Four years of previous inventory efforts using conventional time-constrained search (TCS) methods detected tailed frog at 23/292 discrete sites (7.9% detection rate) in seven watersheds. Non-invasive environmental DNA (eDNA) methods hold promise for cryptic and low-abundance species detection. We rigorously validated a quantitative real-time polymerase chain reaction (qPCR)-based tool for detecting coastal tailed frog eDNA in water samples. This eASTR4 test is highly specific and sensitive. We applied a two-step targeted eDNA analysis approach on duplicate filtered water samples from a total of 72 sites collected over five days. The first IntegritE-DNA step mitigates false negative results and tests all DNA samples for the ability to support amplification from endogenous plant chloroplast DNA as a measure of sample viability. Three DNA samples failed this step even after inhibitor clean up suggesting that these samples were poor quality and not reliable for targeted species’ DNA analyses. All other DNA samples were deemed viable and were then tested for species-specific DNA. Coastal tailed frog eDNA was detected in 55/72 (76%) discrete stream reaches; nine sites with historical known occurrence were all eDNA positive. The false negative rate for TCS compared to eDNA methods was 58%. The results expand known coastal tailed frog distribution to 24 watersheds effectively more than tripling extant occurrences and confirm a previously suspected, apparently isolated coastal tailed frog metapopulation in the Shulaps drainage.

## Introduction

There are two species of tailed frog in the amphibian family Ascaphidae, Rocky Mountain tailed frog (*Ascaphus montanus*) and coastal tailed frog (*A*. *truei*; ASTR), both occurring in British Columbia (BC) [1]. Tailed frogs represent a distinct and ancient lineage. They are unique among anurans as they are associated with mid- to high-elevation mountain streams [2]. Adult, sub-adult, and juvenile (terrestrial) coastal tailed frogs are small, 2.2–5.1 cm snout to vent length, and inconspicuous, appearing on the surface during wet or cool conditions. Tailed frogs have a primitive hopping ability compared to other anurans [3] and their movements are highly localized and restricted to short-distance dispersal; typically remaining within 100 m of their natal streams. Coastal tailed frog adults have been found between 250 to 500 m from perennial streams in old forests [4]. Movements are also likely seasonally restricted as adult frogs are considered to be more susceptible to desiccation than other anurans [5,6]; however, adult females have been reported to migrate between upland habitat and lotic aquatic habitats during the breeding period [7]. Adults are predominantly nocturnal; foraging within riparian habitats along stream edges on a wide array of prey items such as insects, spiders, arthropods, and snails.

**Fig 1:**
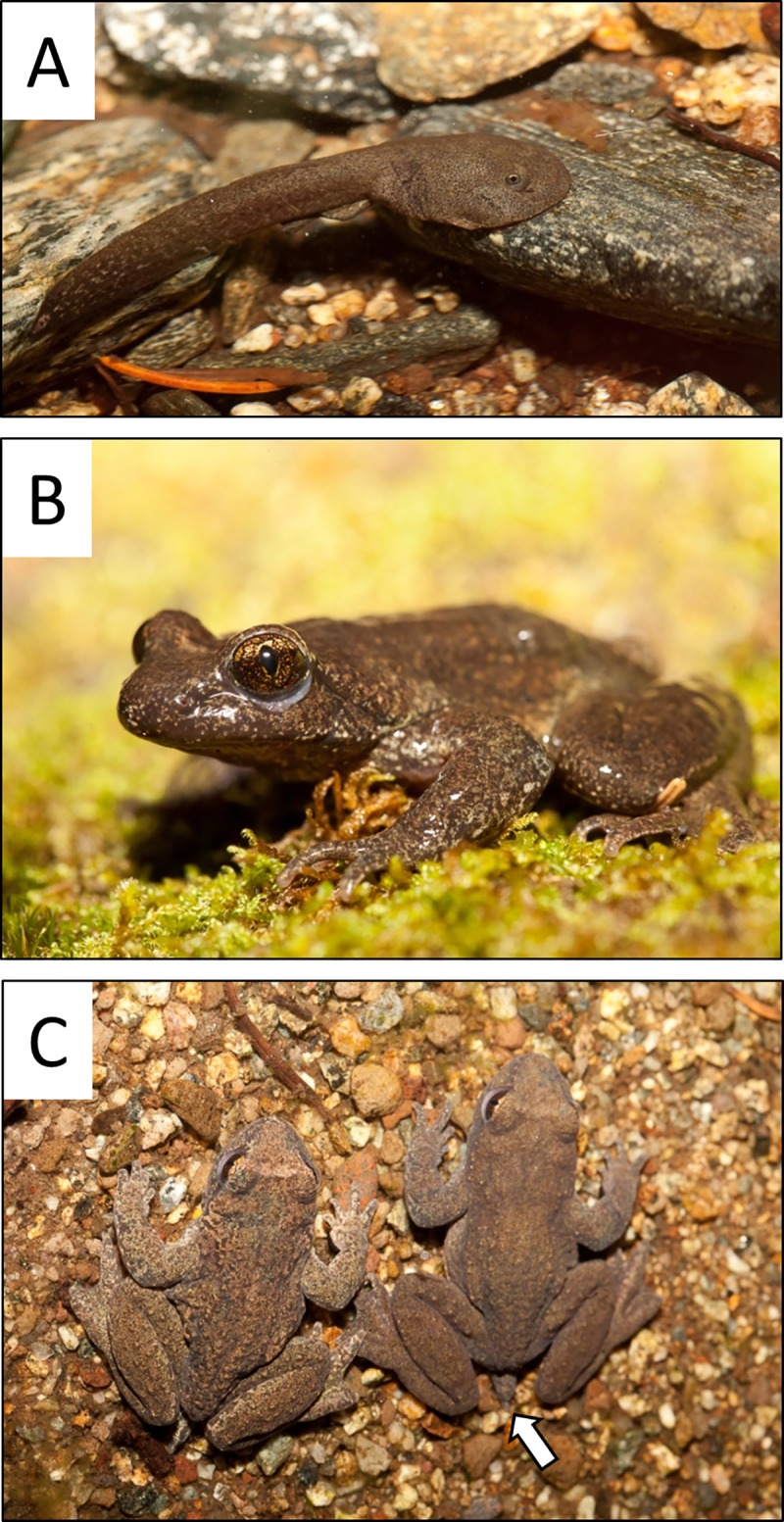
Attributes of coastal tailed frog tadpoles and frogs. A) Coastal tailed frog tadpoles have an adhesive oral-disc, or mouth, to attach to rocks in stream habitats. B) Defining features include, the vertical pupils, lack of an external ‘ear’ membrane, and long outer hind toes. C) Male (right) and female (left) adult tailed frogs are sexually dimorphic–the ‘tail’ is visible on the adult male (white arrow). Photo credits: Jared Hobbs.

Coastal tailed frog breeding occurs in early fall *via* internal delayed fertilization. The following summer the female lays up to 85 colorless eggs, which hatch six weeks later. Embryos feed on yolk sacs through their first winter. By the following spring, the eggs transform into tadpoles with a white spot on their tail (ocellus) that may help distract predators by drawing attention to the waving tail. Tadpoles are approximately 11 mm total length upon hatching and can grow up to 6.5 cm before metamorphosis into a terrestrial form [7]. Tadpoles are also morphologically unique as they possess a large adhesive disk, or sucker, on their anterior ventral surface that aids with foraging in fast-flowing mountain streams (Fig 1A). Tadpoles feed on diatoms that they graze from rocks in both riffle and pool habitats.

Metamorphosis into a frog generally occurs within four years (minimum one year) of hatching [7]. Since maturation to adulthood requires several years, coastal tailed frog populations can only persist in perennial lotic systems [4]. Sub-adults generally reach sexual maturity at eight or nine years of age. Adult frogs have large heads, vertical pupils, no tympana, and broad outer hind toes (Fig 1B). Males have a short ‘tail’ to enable insemination (Fig 1C). This is a necessary adaptation as the more typical anuran method of external fertilization would not be effective in fast-flowing water. These adaptations allow coastal tailed frogs to flourish in cool fast-flowing mountain streams often in isolation from sympatric anurans. This species is long-lived; adults may exceed 20 years of age in the wild [8].

Suitable aquatic habitat occurs within lotic systems that feature a boulder or cobble substrate with abundant interstitial spaces, which provide secure habitat for tadpoles and adults (i.e., refugia from predators and dynamic system events) [4,9]. Occupied streams generally occur in drainages with catchment basins ranging from 0.3–50 km^2^ and stream reaches used for breeding are generally <10 km^2^. Ideal lotic habitats feature step-pool or cascade-pool morphology. Terrestrial forms require mature forests that provide retreat sites (i.e., coarse woody debris) within a stable and moist microclimate as coastal tailed frogs have a narrow temperature tolerance (from 6 to 18°C) [2,4]. Globally, coastal tailed frogs occur along both the west and east side of the Coast and Cascade mountain ranges in North America; from California extending northwards, almost reaching the Alaska Panhandle in the coastal region of northern BC [2,10]. In BC, coastal tailed frogs have a continuous distribution from the international BC/Washington (US) border extending north along the Cascades as far as Lytton (Merritt-Cascades Forest District) and along the Coast Mountain range to at least Kitimat. Its occurrence on the east side of the Coast Mountains is less frequently documented with only a few known extant occurrences near Lytton and a suspected occurrence in the Shulaps. Within the Cascade Mountain range in BC, occurrences in leeward drainages are uncommon [11].

Between the periods of 2000 and 2013, four studies using traditional time constrained surveys (TCS) were performed west of Lillooet, British Columbia in the Cayoosh, Bridge (Shulaps), Seton, Anderson, Carpenter, and Downton Lake drainages; tributaries in the Shuswap Range (i.e., tributaries of the Yalakom River); and around the headwaters of Shulaps Creek (a tributary to the Yalakom River) [12]. The latter two regions had equivocal results that required closer re-evaluation. These studies examined 292 stream reaches over four discontinuous sampling years and found 23 documented coastal tailed frog occurrences (7.9% detection rate; Table 1).

**Table 1:** Detection frequencies of four previous coastal tailed frog TCS project results within the study area. This is a reanalysis and correction of project details presented in [12].

TCS Data Source	Principal Investigator	Year	# of sites	# of occurrences	Detection frequency
Cascades	E. Leupin	2000	44	3	6.8%
Goldbridge-Bralome	Rodway/Regehr	2006	34	1	2.9%
Hurly River	P. Friele/E. Wind	2009	125	7	5.6%
Hurly/Cascades	J. Surgenor	2013	89	12	13.5%
		**Total**	**292**	**23**	**7.9%**

Given the difficult terrain and elusive nature of the coastal tailed frog, we decided to compare TCS with a new environmental DNA (eDNA) method. The term “eDNA” refers to any trace fragment of exogenous DNA that is released by an organism into the environment [13]. The reliable detection of aquatic vertebrate species [14–17], including *Ascaphus montanus* [18,19] using eDNA from a variety of freshwater systems has been previously demonstrated. eDNA has been growing increasingly popular [20] as it is more cost- and time-effective than TCS methods [21].

A critical component to eDNA surveillance methods is the eDNA assay. The assay consists of ex-situ testing for the presence of a species’ DNA using quantitative polymerase chain reaction (qPCR) and it requires the development of a set of species-specific primers and a hydrolysis probe. These primers and probe must be carefully designed and rigorously validated in order to achieve the greatest confidence in the assay [14]. To date, there is no coastal tailed frog-specific eDNA test in the peer-reviewed literature. In order to achieve the objectives of the present study, an eDNA assay was developed and extensively validated for coastal tailed frog to ensure high confidence in test results.

## Materials and methods

### eDNA assay design and validation

TaqMan qPCR primers and probes were developed using mitochondrial gene sequences obtained from the National Center for Biotechnology Information database (https://www.ncbi.nlm.nih.gov). These selected gene sequences included coastal tailed frog as well as sympatric species and human: possible confounders of eDNA detection tools. These gene sequences were aligned using ClustalW (http://www.genome.jp/tools-bin/clustalw) to determine where the sequences share homology. Using the output aln file with BioEdit (Ibis Biosciences, Carlsbad, CA, USA) and Primer Premier Version 6 (Premier Biosoft, Palo Alto, CA, USA) primers and probes were designed for coastal tailed frog targeting unique regions where there was limited identity to the other species in the alignment. Particular care was taken to ensure the designed primers and probes would not cross-react with human. Multiple primer candidates were designed and tested and the successful primer/probe combination is in Table 2. All primers and the probe containing a 5’FAM reporter dye and 3’ ZEN/Iowa Black FQ quencher were ordered from Integrated DNA Technologies (IDT; Coralville, IA, USA).

**Table 2:** Nucleotide sequences for the qPCR-based eASTR4 eDNA tool comprised of primers and a probe for coastal tailed frog detection. The amplicon sequence for the creation of the synthetic DNA sequence is indicated.

Sequence Type	Sequence
Forward Primer	GAACATTGGCATTATCCTACTT
Reverse Primer	AGGCGAAAAATCGTGTTAAC
Probe	FAM-CGCTTTTGTAGGGTATGTGTTACCG-ZEN/Iowa Black FQ
Amplicon	GAACATTGGCATTATCCTACTTTTTCTAGTGATAGCAACCGCTTTTGTAGGGTATGTGTTACCGTGAGGACAAATATCTTTTTGAGGGGCCACTGTAATCACAAATCTTCTCTCTGCTATTCCTTACATCGGAAACGTACTAGTCCAATGGATTTGAGGCGGGTTTTCAGTAGACAACGCCACGTTAACACGATTTTTCGCCT

Total DNA was isolated from confirmed species primarily from roadkill or found dead in the field (Table 3) under Ministry of Forests, Lands and Natural Resource Operations permit #MRNA15-170593 and VI11-71459. All live animals used in the research have been treated humanely according to institutional and national guidelines, with due consideration for the alleviation of distress and discomfort under animal protocol #2015–028 approved by the University of Victoria Animal Care Committee in accordance with the Canadian Council for Animal Care. Amphibia were euthanized with a sodium bicarbonate buffered 0.1% (tadpole) or 1% (frog) tricaine methanesulfonate solution (pH 7.0; Aqua Life, Syndel Laboratories, Nanaimo, BC, Canada). Human DNA was isolated from a HEK293 cell line (American Type Culture Collection (ATCC, Manassas, VA) Catalog number CRL-1573) and used under the University of Victoria Biosafety permit #96876–028. The DNeasy Blood and Tissue Kit (QIAGEN Inc., Mississauga, ON, Canada; Cat# 69506) was used to isolate total DNA from these samples. This DNA was then used to determine the selectivity and sensitivity of the designed primer sets using a CFX96 qPCR machine (Bio-Rad Laboratories, Hercules, CA, USA) following reaction and thermocycling conditions used in [14].

**Table 3:** Names and abbreviations of species used for coastal tailed frog eDNA test validation. The number of technical replicates evaluated was n = 2 except for those indicated in bold where n = 25 technical replicates.

Species Name	Common Name	Species Abbreviation
***Ascaphus montanus***	**Rocky Mountain tailed frog**	**ASMO**
***Ascaphus truei***	**Pacific (coastal) tailed frog**	**ASTR**
***Anaxyrus (Bufo) boreas***	**Western toad**	**ANBO**
***Lithobates (Rana) catesbeiana***	**North American bullfrog**	**LICA**
*Lithobates (Rana) clamitans*	Green frog	LICL
*Lithobates (Rana) pipiens*	Northern leopard frog	LIPI
***Pseudacris regilla***	**Pacific chorus frog**	**PSRE**
***Rana aurora***	**Red legged frog**	**RAAU**
*Rana cascadae*	Cascades frog	RACA
***Rana luteiventris***	**Columbia spotted frog**	**RALU**
*Rana pretiosa*	Oregon spotted frog	RAPR
***Xenopus laevis***	**African clawed frog**	**XELA**
***Taricha granulosa***	**Rough-skinned newt**	**TAGR**
*Ambystoma macrodactylum*	Long-toed salamander	AMMA
*Ambystoma gracile*	Northwestern salamander	AMGR
*Aneides vagrans*	Wandering salamander	ANVA
*Ensatina eschscholtzii*	Ensatina	ENES
*Felis catus*	House cat	FECA
***Homo sapiens***	**Human**	**HOSA**

To determine specificity, the primers were first tested against coastal tailed frog and other species’ total DNA (Table 3) with two technical replicates using a SYBR (Invitrogen, Carlsbad, CA, USA) qPCR assay and agarose gel visualization of the amplified product (amplicon) for the desired species and absence of amplicon in all non-target species. Once the expected 202 bp amplicon was confirmed (Table 2), the primers were then tested in combination with designed hydrolysis probe candidates on the coastal tailed frog DNA plus DNA from 18 other species (two technical replicates). If amplification was detected within 50 cycles (i.e. <50 C_t_) then the replicate was scored as positive.

The best primer/probe set was then further tested if it exhibited specificity for coastal tailed frog. At this stage, total DNA from coastal tailed frog and nine other select species plus human were run with 23 additional technical replicates. Therefore n = 25 technical replicates were run overall.

After specificity testing, sensitivity was initially determined using a five-fold dilution series of the coastal tailed frog total DNA between 0.008 and 5 μg/L. Twenty-five technical replicate qPCR reactions were run for each DNA concentration in addition to the no DNA template negative control. The binomial standard error was calculated as in [14].

The sensitivity of the eASTR4 qPCR test was further empirically evaluated using a synthetic DNA fragment and methods adapted and modified from [22]. This method is most conducive for standardization and inter-laboratory comparison as it relies upon the use of an easily accessible source of synthetic DNA that can readily be prepared according to copy number to assess assay performance. The double-stranded synthetic gBlocks DNA was ordered from IDT and consisted of the 202 bp amplicon sequence in Table 2.

In order to stabilize the synthetic DNA and its subsequent dilutions in solution, we prepared a stock solution of tRNA (Sigma-Aldrich Canada Co., Oakville, ON, Canada; Cat# 10109495001) in diethylcarbonate (DEPC)-H_2_O (Thermo Fisher Scientific Inc., Ottawa, ON, Canada; Cat# AM9922). This tRNA stock was stored in aliquots at -20°C before use. The working tRNA solution was prepared immediately before use by performing a 10x dilution of 100 ng/μL tRNA in Tris-EDTA Buffer pH 8.0 (Thermo Fisher Scientific Inc., Ottawa, ON, Canada; Cat# AM9858).

The lyophilized synthetic DNA was suspended in an appropriate volume of working tRNA solution to create a 10^10^ copies/μL superstock. This superstock was then diluted to 10^7^ copies/μL using working tRNA solution. One μL of this dilution was added to 31 μL of working tRNA solution to produce a temporary stock containing 312,500 copies/μL.

This stock was then serially diluted five-fold to produce a range of ten synthetic DNA concentrations from 31,250 copies/μL to 0.016 copies/μL. Two μL of each dilution were run in qPCR reactions with eight technical replicates using the same reaction conditions as [16]. Therefore the final range tested per reaction was 0.032 to 62,500 copies per reaction. The resulting data were plotted against the copy number per reaction to determine the limits of detection (LOD) and quantitation (LOQ). The LOQ was defined as the lowest standard dilution that amplified in 90% of the replicates [22] and the LOD is the lowest copy number at which DNA is detected above background.

### Study area and sample site selection

The Bridge River and Seton, Anderson, Carpenter, and Downton Lake drainages (approximately 3,700 km^2^) are located in the xeric rain-shadow of the southern coastal mountains. The study area is located in the Interior Transition Ranges ecoregion within the Southern Interior Ecoprovince [23].

Lower elevations within the study area occur within the Interior Douglas-Fir (IDF) bio-geoclimatic (BEC) zone and are characterized by warm, dry summers, a fairly long growing season, and cool winters [23]. The Coastal Western Hemlock (CWH) BEC zone occurs at low to mid-elevations (up to 990 m above sea level (ASL)) in the study area. The CWH has the highest level of annual rainfall of all the BEC zones in the study area with a cool meso-thermal climate (cool summers and mild winters) [23].

Upper elevation habitats occur within the Montane Spruce (MS) and Engelmann Spruce-Sub-alpine fir (ESSF) BEC zones before transitioning into the Interior Mountain-heather Alpine (IMA) BEC zone [23]. The ESSF BEC zone occurs at elevations between 900–2100 m ASL (lower elevation limits vary by aspect), and is characterized to have a severe climate with long cold winters and short cool summers. The MS BEC zone occurs at mid-elevations in the central interior, on the leeward side of the Coast Mountains. Cold winters and short warm summers are characteristic in these areas; forested areas are dominated by sub-alpine fir (*Abies lasiocarpa*). Coastal tailed frog is commonly occurs in the MS, CWH, and ESSF BEC zones, and occurs with lower reported frequency in the IDF BEC zone.

Seventy-two sites were selected based on the consideration of several criteria including results from previously applied TCS methods. As these sites were on Crown Land, no permits were necessary. Forty-five eDNA sampling sites were within 2 km of TCS sites documented in one or more of the four previous surveys within the study area (Table 1). Nine of these had documented positive coastal tailed frog detection and were included during sample design and collection to allow comparison of methodological efficacy with the eASTR4 eDNA test at known extant coastal tailed frog sites. Consideration of surficial geology, catchment size, and origin (lake fed *versus* glacier fed) was also included to ensure representation of habitat heterogeneity in sample site placement. Details regarding the site attributes are presented in S1 Table.

### Collection and filtration of water samples

One hundred and forty-four water samples were collected from 72 sites between August 13 and 18, 2016. Duplicate 1 L water samples were collected in polypropylene bottles at each sample location or ‘site’. No permits were necessary for water collection on Crown Land. Sites near a stream confluence were sampled (~50 m) upstream of the confluence to ensure samples were not contaminated by water from effluent immediately downstream and eliminate ambiguity regarding eDNA source. Each sample was collected as close to the thalweg of the stream as possible, as thalwegs concentrate particulate matter, including DNA, into a narrow stream channel; thereby theoretically raising the probability of a positive test if the targeted species is present [18]. To limit degradation of DNA in each sample, collected samples were placed in an insulated cooler, in contact with ice, immediately following collection until they were filtered. All samples were filtered within 24 h of collection using a vacuum pump and 47 mm diameter cellulose nitrate filters (0.45 μM pore size; Thermo Fisher Scientific Inc., Ottawa, ON, Canada; Cat# N1452045). Following filtration, the membrane was placed into a 2 mL sterile polypropylene cryogenic vial filled with 95% molecular-grade ethanol. A single 1 L control sample (i.e., distilled water) was processed for each day of sample collection using the same filtration protocol as the site water to serve as a control for the collection, filtration, and laboratory extraction and analysis processes.

### Isolation of DNA from filter membranes

**Fig 2:**
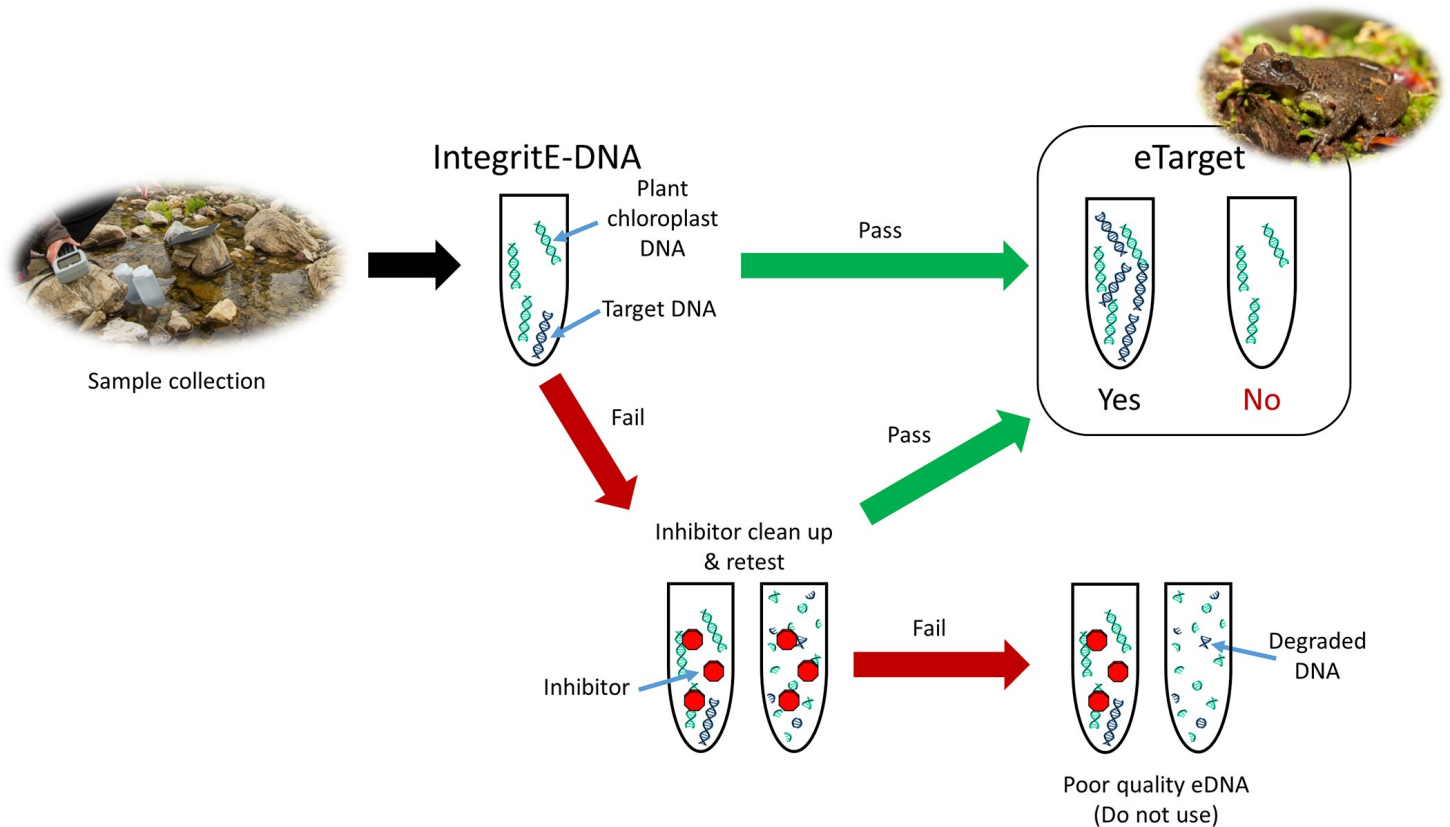
Use of the IntegritE-DNA test leads to increased confidence of species-specific DNA (eTarget) results. The IntegritE-DNA test evaluates the ability of the isolated DNA in a sample to amplify plant chloroplast DNA prior to testing for the target species. If the sample passes the IntegritE-DNA test, then it is then tested for the target species (eTarget). If a sample fails the IntegritE-DNA test, it undergoes clean-up to remove inhibitors before being retested. If the cleaned up sample fails the IntegritE-DNA test, it is deemed poor quality due to either degraded DNA or retention of inhibitors and the sample is not considered reliable. Photo credits: Jared Hobbs.

DNA was isolated from the “A” replicate samples from each site and tested first. If the sample passed the two-step evaluation process described below (Fig 2) and resulted in an eASTR4 test score of ≥3/8 positive hits [14], then the site was automatically assigned a positive target eDNA detection for the site and the “B” replicate was not evaluated. Otherwise, the “B” replicate was subsequently evaluated using the two-step evaluation process as well.

Prior to isolating DNA from the filter membranes, the filter samples were randomized and assigned a DNA Processing Number (DPN) to eliminate processing bias. DNA was isolated using an established protocol [14] with the following modifications: Following overnight digestion, the sample was transferred to a Qiashredder column (QIAGEN Inc., Mississauga, ON, Canada; Cat# 79656) and the eluate from this column was carried through the remaining DNeasy Blood and Tissue kit protocol. All DNA isolations and eDNA assay set-up was conducted in a laminar flow hood with a HEPA filter. To eliminate the chances of a contamination event occurring, the workspace was cleaned with a 10% bleach solution prior to use and dedicated electronic pipettes with filter tips were used.

### eDNA assay set-up and data analysis

Prior to analyzing each sample for the target taxa, an “IntegritE-DNA” DNA viability control was run to ensure the quality of DNA extracted was suitable for qPCR analysis and that there were no inhibitors present (Fig 2). To test this, four technical replicates of the ePlant5 qPCR assay were run as described in [14]. If amplifiable DNA was confirmed in the sample, we proceeded on the knowledge that inhibition was not impeding the qPCR amplification process for that sample and that eDNA of sufficient quality had been successfully captured during filtration. If the ePlant5 test was negative, the sample was further processed using the Zymo OneStep PCR Inhibitor Removal Kit (Cedarlane, Burlington, ON, Canada; Cat# D6030S) and retested. If the sample still yielded a negative result, then that sample failed quality control due to inhibition or degradation and was not further tested or included in the data set (Fig 2). This control step is essential to eliminate mistaken assignation of a negative result.

**Fig 3:**
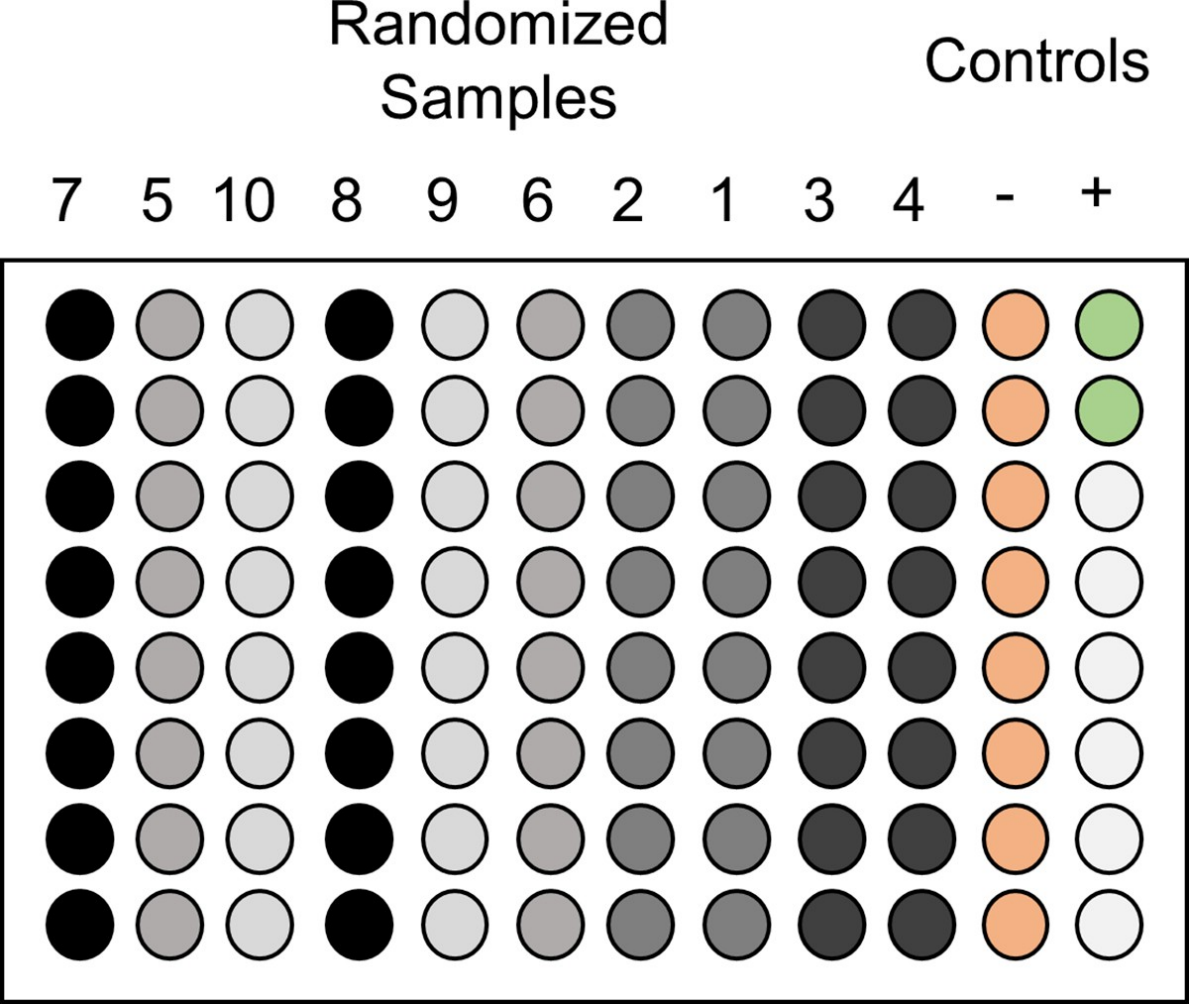
Sample orientation on qPCR plates plays an important role in the success of the eDNA assay. eDNA samples are randomized before DNA is isolated and the randomized samples are run in eight technical replicates for the eASTR4 test. Using the above layout spatially removes the positive control from the test samples to limit to chance of contaminating the test samples with pure target species total DNA. If a contamination event has occurred it will be detectable in the negative control right adjacent to the positive control.

Each isolated DNA sample that passed the IntegritE-DNA test was then assessed using the eASTR4 test using eight technical replicate qPCR reactions with each qPCR plate including two positive and eight negative PCR reaction controls (Fig 3). The positive controls contained coastal tailed frog total DNA and the negative controls contained no DNA template with the missing reaction volume replaced with ultrapure water. The configuration of the qPCR plates was designed to have the positive controls spatially removed from the samples to reduce the likelihood of contamination. The negative control reactions separate the test samples from the positive controls so if a contamination event occurred it would be detected in the negative controls (Fig 3).

## Results

### Coastal tailed frog eDNA test design and validation

**Fig 4:**
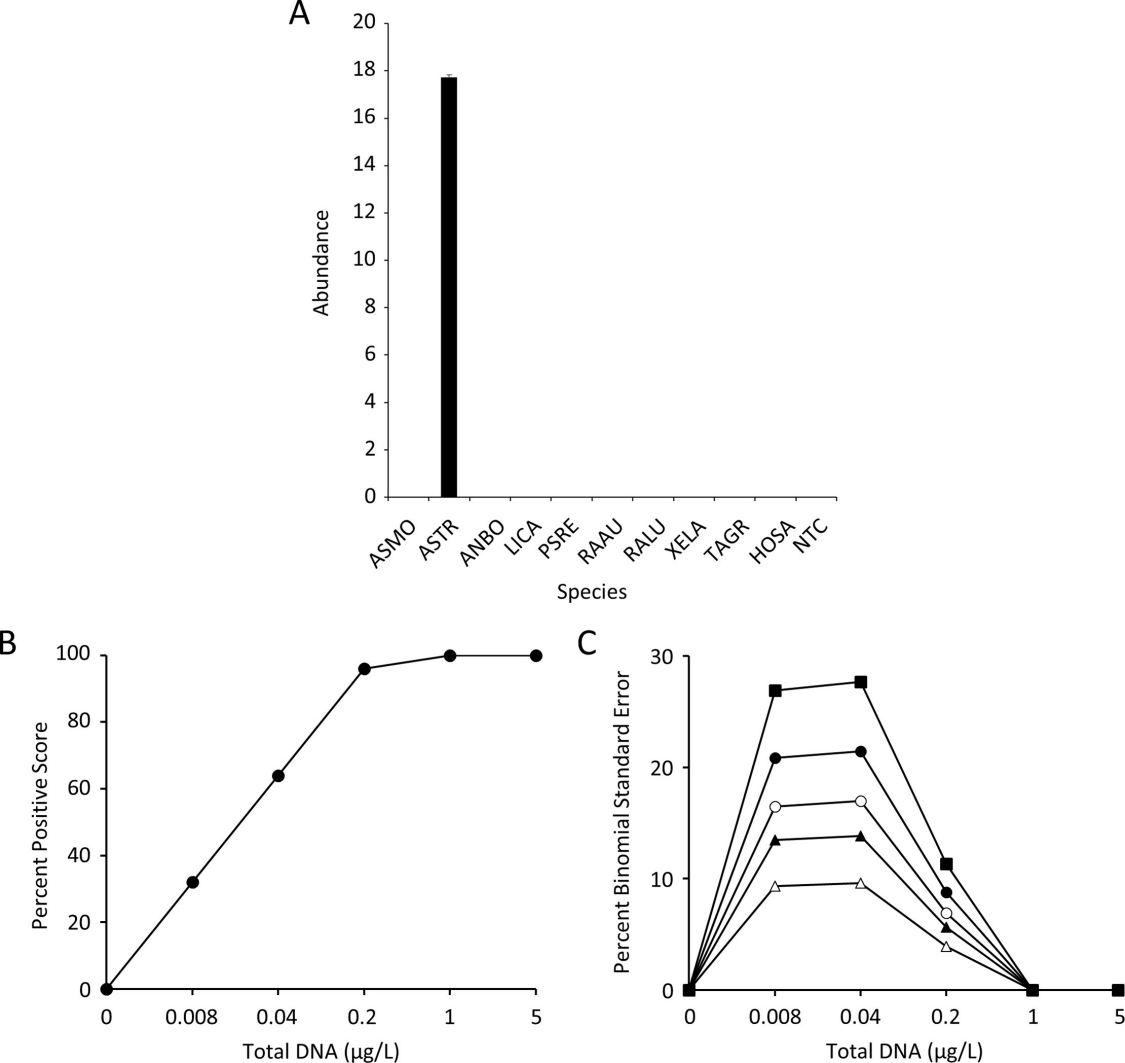
The eASTR4 test is highly specific for coastal tailed frog and is highly sensitive. A) The sensitivity graph is displayed as mean abundance with standard error of the mean of n = 25 technical replicates of each species (refer to Table 1 for legend) and a no template control (NTC). Five μg/L total DNA were tested and the strength of detection (Abundance) was determined as the number of cycles (50) in the qPCR program minus the cycle threshold where detection occurred. B) The sensitivity graph was produced using a 5-fold dilution series of total DNA of the target species. The percent positive score is derived from the number of detected reactions divided by the number of technical replicates (n = 25). C) Percent binomial standard error determined at each dilution of total DNA for differing technical replicates: n = 3 (solid squares), n = 5 (solid circles), n = 8 (open circles), n = 12 (solid triangles), and n = 25 (open triangles).

A qPCR tool was successfully designed for coastal tailed frog detection after consideration of multiple primer/probe combinations derived from the mitochondrially encoded 12S RNA (MT-RNR1), 16S RNA (MT-RNR2), NADH:ubiquinone oxidoreductase core subunit 1 (MT-ND1) and cytochrome B (MT-CYB) genes. This tool is designated as eASTR4 and targets a 202 bp amplicon from the MT-CYB gene; see Table 2 for oligo sequences. The eASTR4 test is highly specific to coastal tailed frog detecting with 100% frequency at both 5 μg/L and 1 μg/L total DNA and with excellent strength of detection (Fig 4A). None of the other species were detected by the eASTR4 test. This tool is highly sensitive for the target species with detection at the lowest DNA dilution tested (0.008 μg/L; Fig 4B). As expected, the highest binomial standard error for this tool is at those concentrations where the percent positive score is between 0 and 100% (Fig 4C). We examined the effect of the number of technical replicates on this variation and found that the highest binomial standard error was 28% with three technical replicates. This variation decreased as the number of technical replicates increased to 22% (n = 5), 17% (n = 8), 14% (n = 12), and 10% (n = 25; Fig 4B). Eight reactions were determined to provide an appropriate balance between effort, cost, and reasonable binomial error based upon power [14].

**Fig 5:**
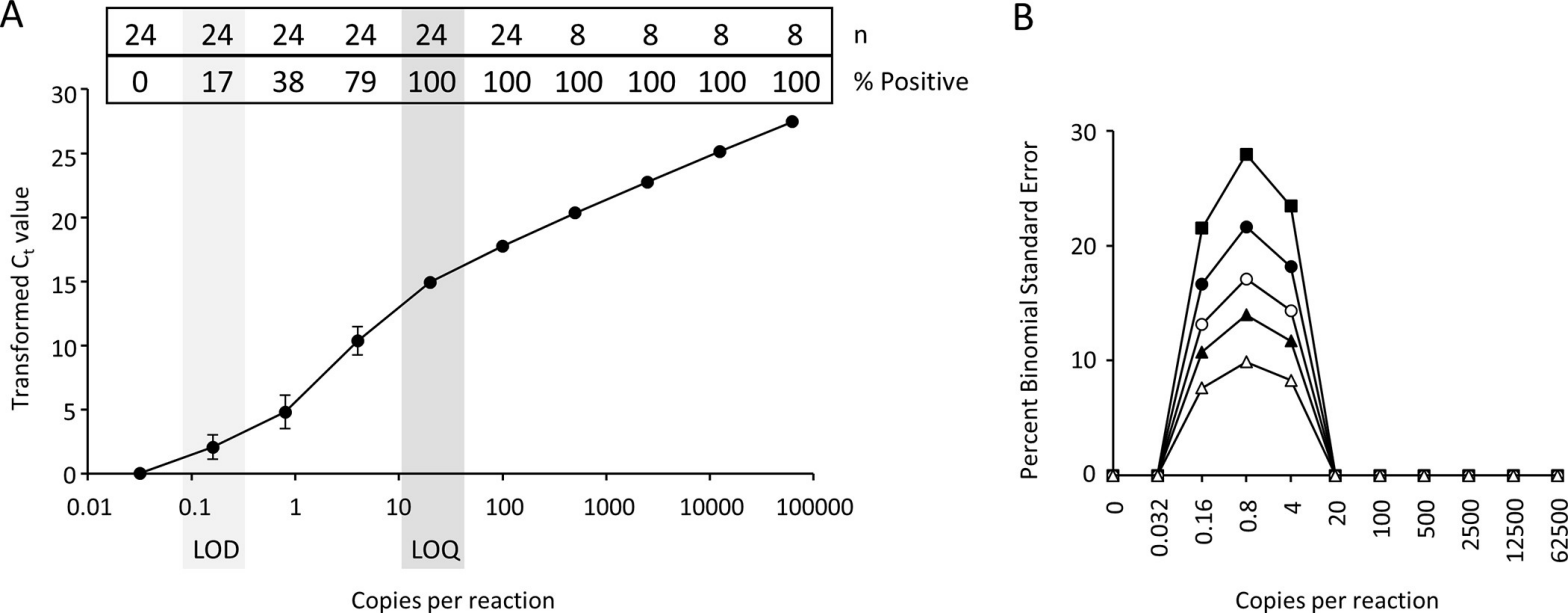
Sensitivity and determination of the limits of detection (LOD) and quantitation (LOQ) for the eASTR4 test using synthetic template DNA. A) Five-fold serial dilutions of a synthetic template DNA were added to the eASTR4 reaction at copy numbers ranging from 62,500 to 0.032 copies per reaction and the mean transformed C_t_ values (50.001-C_t_) determined for each of the eight or 24 technical replicates. The whiskers depict the standard error of the mean. The NTC controls never had any amplification detected. The proportion of technical replicates that passed the C_t_ cut off for detection (% Positive) and number of technical replicates (n) measured are indicated at the top of the graph for each dilution tested. B) Percent binomial standard error determined at each dilution of synthetic template DNA for differing technical replicates: n = 3 (solid squares), n = 5 (solid circles), n = 8 (open circles), n = 12 (solid triangles), and n = 25 (open triangles).

Analysis of the dilution curves generated with the synthetic DNA template revealed the eASTR4 tool to have consistent and reliable detection (Fig 5A). The LOQ was determined to be 20 copies/reaction with 100% positive hits out of 24 technical replicates and the LOD with 24 technical replicates was at 0.16 copies/reaction (Fig 5A). This means that if 100 technical replicates were run using binary detection, 16 positive hits would be expected. This is also comparable to a 1/8 level of detection used within the present study for the field samples. The binomial standard error calculated with the synthetic DNA was identical to that determined for total DNA (compare Figs 4C to 5B) suggesting that the reaction conditions are optimal and that the use of synthetic DNA is a suitable surrogate for total DNA isolated from tissue.

To date, we have run 265 no template controls (NTCs) in the context of 11 separate projects with 129 directly relating to the eASTR4 test validation and the present study. We have never had a positive hit in any of these technical replicates demonstrating that the background noise for this test is zero.

### Field site coastal tailed frog occurrence

All isolated eDNA samples were subjected to the IntegritE-DNA test. We have experienced a consistent background level of 36.32±0.15 (n = 56) from the ePlant5 tool within this study and previously [14]. For this reason, we set a cut off of ≤30 C_t_ for identifying a good quality sample [14]. The average C_t_ from 117 field sample filters was 22.1±0.18. Three samples had C_t_ values >30, thus failing the IntegritE-DNA test (S2 Table). These samples were subjected to an additional inhibitor clean-up step and retested (Fig 2). They still failed suggesting degraded DNA in the sample, so these samples were deemed poor quality and thus the results from these samples are prone to false negatives and are unreliable.

Keeping the results of the serial dilutions of total and synthetic DNA in mind and the observation that all of the distilled water field sample controls returned 0/8 for the eASTR4 test, we analyzed and interpreted the eASTR4 test results for each sample as follows. A positive result on a sample was assigned if ≥1 (of 8) technical replicates returned a positive result (C_t_ <50). A site was deemed positive if either of the two replicate samples yielded a positive qPCR score.

**Fig 6:**
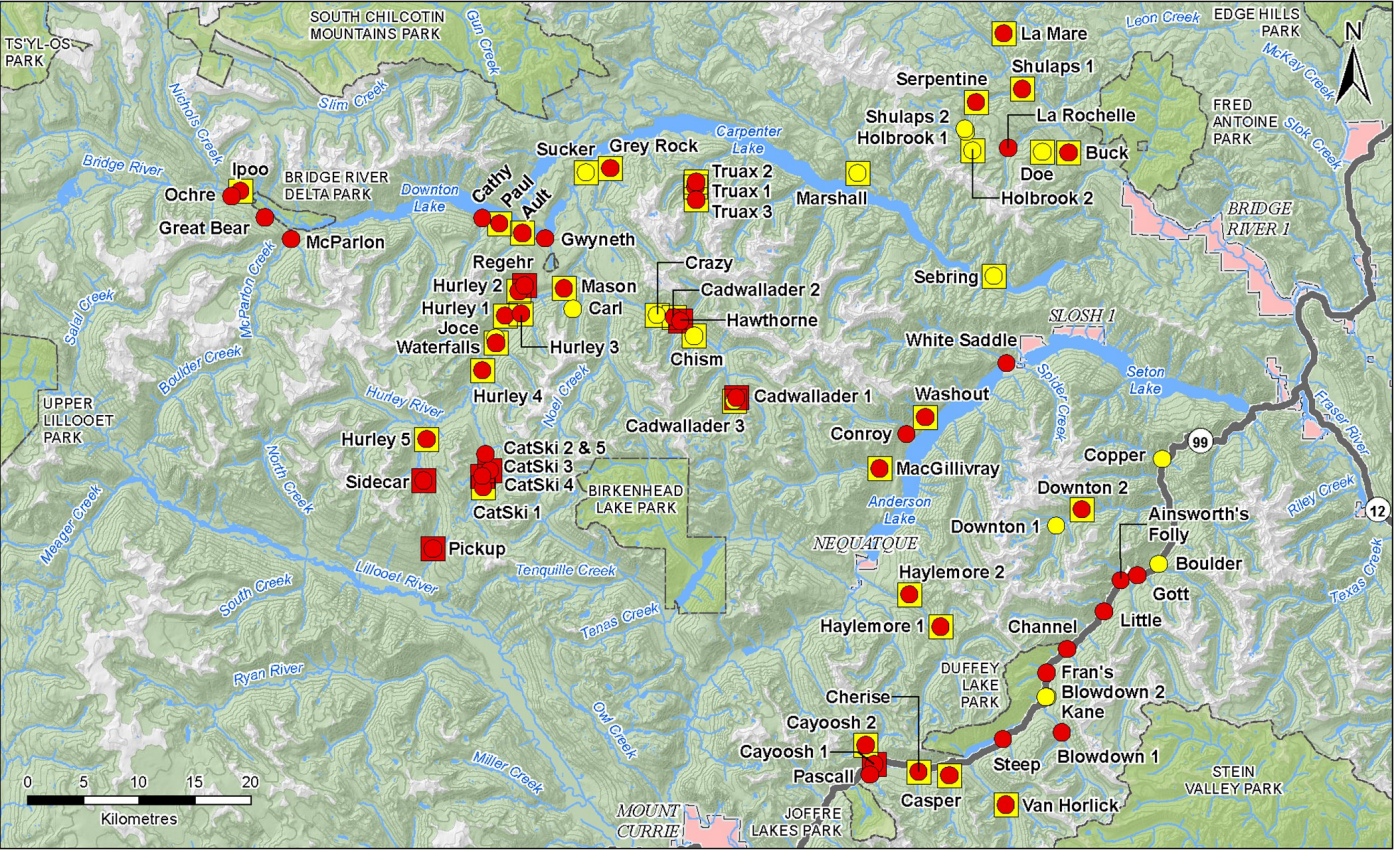
Fifty-five of 72 sampling sites located in British Columbia’s southern coastal mountains were positive for coastal tailed frog using eDNA methods. The sampling sites were located in Bridge River and Seton, Anderson, Carpenter and Downton lake drainages. Squares indicate sites that were assessed by previous time constrained surveys and circles indicate the eDNA results from the same sites. Squares, historical TCS results from the studies in Table 1; Circles, eDNA results; Red, positive detection; Yellow, negative detection. The map was created from open source material from the governments of Canada and British Columbia (https://www2.gov.bc.ca/gov/content/data/open-data/open-government-license-bc; https://open.canada.ca/en/open-government-licence-canada).

Fifty-five of the 72 (76%) sites tested positive, resulting in confirmation of coastal tailed frog DNA within all higher-order watersheds in the study area (Fig 6). Details regarding the relationship between historic TCS results with eDNA results are presented in S3 Table. Of the 72 sites tested, nine sites were known to support coastal tailed frog based on results from previous TCS surveys (Table 4). All of these known sites tested positive using the eDNA methods in the present study (Table 4) with the most concentrated DNA samples slightly below the LOQ with an estimated 5 copies/reaction. Thirty-six sites had previously returned negative historical TCS results within a 2 km radius (Table 4). Of these, 21 tested positive for coastal tailed frog eDNA (Table 4). Thus the false negative rate for the TCS method is calculated to be 58% (Table 4). Although outside the scope of the current historical study comparators, the veracity of these new positive sites is supported by two independent observations of coastal tailed frogs at Blowdown 1 in 2015 and 2016 (P. Friele and F. Iredale, personal communication) and an observation at the Channel site in 2015 (F. Iredale, personal communication).

**Table 4:** Relationship between coastal tailed frog historical TCS and eDNA results. The number of positive and negative sites where both TCS and eDNA results were within a 2 km radius are shown.

Type of detection method	Positive		Negative		Total
TCS	9		36		45
	**Positive**	**Negative**	**Positive**	**Negative**	
eDNA	9	0	21	15	45
Concordance	100%		58%	42%	

Four BEC zones were sampled including: CWH (5 sites), ESSF (17 sites), IDF (23 sites) and MS (27 sites) (Fig 6). Coastal tailed frog eDNA was detected at six sites in CWH (100% of sites sampled), 13 sites in ESSF (81% of sites sampled), 17 sites in IDF (74% of sites sampled) and 19 sites in MS (70% of sites sampled). The sampling sites were at an elevation between 255–1758 m ASL and coastal tailed frog eDNA was only detected in sites from 470–1513 m ASL. Of the 28 watersheds sampled, coastal tailed frog eDNA was detected in 24 of them. More precise site-level considerations are not synthesized here as eDNA transport in lotic systems is inevitable. As such, analysis of sample site characteristics does not necessarily confer meaningful or representative information regarding coastal tailed frog habitat characteristics [22].

## Discussion

eDNA methods are becoming increasingly popular as a surveillance tool, particularly for application on species that are difficult to detect using traditional surveillance methods. These include species with low population densities, inconspicuous species with secretive ecologies (i.e., nocturnal, cryptic or well-concealed) and species that occupy habitats that are difficult to survey [22].

Despite advances being made to improve rigor and ensure consistency in laboratory methods and interpretation, there still is a dire need for improved and consistent application of eDNA techniques [14,20,24]. False negative detection is a particular issue with species that are cryptic and occur at low densities. This has led some to utilize statistical methods based upon site occupancy models to estimate detection probabilities [25,26]. eDNA studies typically only test for enzyme inhibition in laboratory tests using exogenous “internal positive control (IPC)” DNA that is added to a qPCR reaction. While effective at detecting inhibitors co-isolated with the DNA from environmental samples, the IPC is not able to determine the integrity of the DNA sample (i.e. DNA degradation) [14]. Thus use of an IPC could still lead to a false negative result. The present study adds a rigorously field- and laboratory-validated coastal tailed frog eDNA detection tool to the arsenal of eDNA methods and demonstrates the utility of using an IntegritE-DNA test for assessing sample quality in tandem with this new coastal tailed frog eDNA test. This combinatorial approach lends itself to better quality empirical data and interpretation than IPC-based methods.

The TCS detection probability within the context of the present study was enhanced substantially by approximately an order of magnitude using eDNA methods for detecting coastal tailed frog eDNA. The study area was particularly conducive to such a comparison given the challenging terrain and high likelihood of coastal tailed frog presence. Other studies on amphibians have realized enhanced detection probabilities; the magnitude of which was most influenced by the method of detection used; gel-based, semi-quantitative methods perform less well than qPCR-based methods [14,18,27,28].

Prior to the present application of eDNA in the Bridge River system, there had been four extensive previous studies over a 13 year period using conventional methods to document presence and distribution of coastal tailed frog within the study area (Table 1). We collected stream water samples from the same 28 previously sampled watersheds over the course of five consecutive days at a fraction of the cost and effort expended with TCS methods, and with a much-improved detection rate. Coastal tailed frog eDNA was detected at 55 sites increasing the number of confirmed extant coastal tailed frog occurrences to 24 watersheds within the study area. Results from the eDNA survey provide evidence of coastal tailed frog occurrence in the headwaters of the Downton and Anderson Lake watersheds, and provide conclusive evidence of an extant coastal tailed frog population in the Shulaps range, along the west-side of the Yalakom River drainage. Additional occupied tributaries in each of the drainages where coastal tailed frog was known to occur were also documented in the present study. The eDNA results are likely reflective of current coastal tailed frog occupying the streams and immediate environs as eDNA typically persists for a few weeks and does not generally travel more that a few kilometers (reviewed in [22]).

The results of the present study, when compared to results from previous studies using conventional methods, provide compelling support for the use of eDNA as an alternative method for detecting the presence of aquatic taxa, including coastal tailed frog. The rapid field collection associated with eDNA studies (relative to conventional TCS methods), the relatively low cost of filter materials, the elimination of observer bias, and relatively high efficacy (i.e., relatively greater detection probabilities) suggest that eDNA methods are more efficient and more effective for coastal tailed frog inventory than standard TCS methods.

## Supporting information

S1 Table Location and attributes of the present study sites sampled for coastal tailed frog eDNA analysis. (PDF)

S2 Table Individual sample results for the IntegritE-DNA and eASTR4 tests. IntegritE-DNA tests were performed in four technical replicates and the eASTR4 test was done in eight technical replicates. Samples that failed the IntegritE-DNA test are indicated in orange.
(PDF)

S3 Table Comparison of historical TCS results with eDNA results for coastal tailed frog. Historical data from four previous surveys within the region of interest (see Table 1 for details) were paired with eDNA sampling sites when the recorded location was within 2 km of the sampling site.
(PDF)
