# Supplementary material for: Expansion of the known distribution of the coastal tailed frog, Ascaphus truei, in British Columbia, Canada, using robust eDNA detection methods
Source: PLoS One. 2019 Mar 14;14(3):e0213849. doi: 10.1371/journal.pone.0213849 (PMC6417668; doi:10.1371/journal.pone.0213849)
Supplement: S1 Table — (PDF) [file pone.0213849.s001.pdf]

S1 Table. Location and attributes of the present study sites sampled for coastal tailed frog eDNA analysis.

Location Name	Collection date	Zone	Easting	Northing	Elevation (± 5 m)	Time	Precipitation	Cloud Cover (%)	Air Temperature (ºC)	Water Temperature (ºC)	Stream Order	Dominant Substrate	Subdominant Substrate	Embeddedness	Logging Activity
Ainsworth's Folly	2016-08-16	10	559809	5597971	891	16:00	None	0	25	13	S3	Cobble	Gravel	Low	No
Ault	2016-08-14	10	506237	5629120	675	14:30	None	0	30	-[a]	S3	Fractured bedrock	Cobble	Medium	No
Blowdown 1	2016-08-16	10	554572	5584413	1367	14:00	None	0	28	14	S2	Boulder	Gravel	Medium	Yes
Blowdown 2	2016-08-16	10	553243	5587584	1176	15:00	None	0	25	14	S6	Cobble	Boulder	Low	Yes
Boulder	2016-08-16	10	563219	5599470	825	16:40	None	0	30	14	S3	Boulder	Gravel	Low	No
Buck	2016-08-17	10	555163	5636258	-	16:40	None	0	30	11	S2	Boulder	Cobble	Low	Yes
Cadwallader 1	2016-08-14	10	525408	5614376	1435	16:45	None	0	25	-	S3	Boulder	Gravel	High	Yes
Cadwallader 2	2016-08-14	10	519817	5621578	1286	17:31	None	0	23	-	S6	Cobble	Gravel	Medium	Yes
Cadwallader 3	2016-08-14	10	525243	5614052	1420	16:35	None	0	25	-	S3	Cobble	Fines	High	Yes
Carl	2016-08-15	10	510729	5622334	995	12:00	None	0	20	-	S3	Cobble	Gravel	Low	Yes
Casper	2016-08-16	10	544453	5580549	1255	11:55	None	0	25	-	S3	Cobble	Gravel	Low	Yes
Cathy	2016-08-14	10	502682	5630448	799	14:05	None	0	30	-	S3	Boulder	Gravel	Medium	No
CatSki 1	2016-08-13	10	502739	5606331	1365	16:05	None	0	26	12	S6	Fines	Gravel	High	Yes
CatSki 2	2016-08-13	10	502942	5609315	1305	16:50	None	0	23	-	S2	Fines	Cobble	High	No
CatSki 3	2016-08-13	10	503308	5607852	1354	16:37	None	0	25	10	S3	Boulder	Fines	Medium	Yes
CatSki 4	2016-08-13	10	502711	5607302	1387	16:25	None	0	26	8	S3	Fines	Boulder	High	Yes
CatSki 5	2016-08-13	10	502917	5609316	1305	17:00	None	0	20	6	S6	Gravel	Fines	High	Yes
Cayoosh 1	2016-08-16	10	537721	5581574	1269	11:00	None	0	25	11	S2	Cobble	Gravel	Medium	No
Cayoosh 2	2016-08-16	10	536987	5583234	1359	10:20	None	0	20	10	S3	Gravel	Cobble	Medium	Yes
Channel	2016-08-16	10	555031	5591837	1033	15:30	None	0	25	16	S3	Boulder	Gravel	Low	No
Cherise	2016-08-16	10	541719	5580823	1243	11:20	None	0	22	9	S2	Cobble	Gravel	High	No
Chism	2016-08-14	10	521597	5619908	1314	17:15	None	0	23	-	S5	Cobble	Gravel	High	No
Conroy	2016-08-17	10	540654	5611093	470	15:30	None	0	28	14	S2	Cobble	Boulder	Low	No
Copper	2016-08-16	10	563488	5608826	659	16:45	None	0	30	10	S3	Cobble	Gravel	Low	No
Crazy	2016-08-14	10	518305	5621718	1271	17:45	None	0	23	-	S3	Cobble	Gravel	Low	Yes

Doe	2016-08-17	10	552806	5636374	1412	16:30	None	0	30	8	S2	Cobble	Gravel	High	Yes
Downton 1	2016-08-17	10	554056	5602877	1475	20:50	None	0	30	10	S3	Boulder	Cobble	Low	Yes
Downton 2	2016-08-17	10	556348	5604314	-	20:30	None	0	30	10	S3	Boulder	Cobble	Low	Yes
Fran's	2016-08-16	10	553144	5589725	1062	15:15	None	0	25	12	S3	Cobble	Gravel	Low	No
Gott	2016-08-16	10	561321	5598445	833	16:30	None	0	30	16	S2	Cobble	Gravel	High	No
Great Bear	2016-08-14	10	483205	5630527	996	13:00	None	0	30	9	S3	Boulder	Gravel	Low	Yes
Grey Rock	2016-08-15	10	514123	5634913	887	7:50	None	0	12	9	S2	Gravel	Fines	High	Yes
Gwyneth	2016-08-14	10	508268	5628612	888	14:45	None	0	30	-	S6	Boulder	Cobble	Medium	No
Hawthorne	2016-08-14	10	520410	5621247	1279	17:26	None	0	23	-	S2	Boulder	Gravel	High	Yes
Haylemore 1	2016-08-17	10	543713	5593884	1103	17:44	None	0	30	11	S5	Boulder	Cobble	Low	No
Haylemore 2	2016-08-17	10	540901	5596703	837	18:10	None	0	30	15	S5	Cobble	Gravel	Medium	Yes
Holbrook 1	2016-08-17	10	545974	5638199	1453	15:30	None	0	30	11	S3	Cobble	Gravel	High	No
Holbrook 2	2016-08-17	10	546566	5636501	1758	15:45	None	0	30	12	S3	Boulder	Cobble	Medium	Yes
Hurley 1	2016-08-15	10	504738	5621701	1072	13:15	None	0	25	-	S3	Gravel	Boulder	Medium	Yes
Hurley 2	2016-08-15	10	505892	5623907	1152	13:00	None	0	25	-	S6	Cobble	Gravel	High	Yes
Hurley 3	2016-08-15	10	506092	5621859	1034	12:30	None	0	25	-	S3	Cobble	Boulder	Low	Yes
Hurley 4	2016-08-13	10	502705	5616781	1073	17:45	None	0	27	10	S3	Cobble	Gravel	High	Yes
Hurley 5	2016-08-13	10	497642	5610606	1202	15:12	None	0	28	8	S6	Cobble	Gravel	High	Yes
Ipoo	2016-08-14	10	481016	5632853	866	12:32	None	0	30	9	S6	Boulder	Fines	Medium	No
Joce	2016-08-15	10	503823	5619707	1035	13:40	None	0	30	-	S6	Cobble	Gravel	Medium	No
Kane	2016-08-16	10	553085	5587549	1117	13:20	None	0	25	14	S2	Cobble	Gravel	Medium	Yes
La Mare	2016-08-17	10	549345	5646965	869	12:05	None	0	23	8	S3	Gravel	Fines	Low	No
La Rochelle	2016-08-17	10	549711	5636705	1513	16:45	None	0	30	11	S3	Cobble	Gravel	Medium	Yes
Little	2016-08-16	10	558306	5595181	979	15:45	None	0	25	12	S3	Cobble	Gravel	Low	No
MacGillivray	2016-08-17	10	538211	5608006	664	16:06	None	0	28	12	S2	Boulder	Fines	Medium	No
Marshall	2016-08-17	10	536264	5634411	824	11:16	None	0	25	9	S3	Cobble	Gravel	Low	No
Mason	2016-08-15	10	509954	5624130	978	12:15	None	0	22	-	S3	Cobble	Gravel	Low	Yes
McParlon	2016-08-14	10	485540	5628535	900	13:30	None	0	30	8	S2	Boulder	Gravel	High	No
Ochre	2016-08-14	10	480227	5632435	866	12:41	None	0	30	9	S6	Boulder	Gravel	Low	No
Pascall	2016-08-16	10	537358	5580586	1237	9:10	None	0	16	8	S3	Cobble	Boulder	Low	Yes

Paul	2016-08-14	10	504232	5629919	792	14:15	None	0	30	-	S3	Cobble	Gravel	Medium	No
Pickup	2016-08-13	10	498236	5600828	993	14:20	None	0	30	9	S6	Gravel	Cobble	Low	Yes
Regehr	2016-08-15	10	506427	5624391	1179	12:50	None	0	25	-	S6	Boulder	Gravel	Low	Yes
Sebring	2016-08-18	10	548481	5625237	661	14:35	Light rain	100	15	-	S5	Fines	Gravel	High	No
Serpentine	2016-08-17	10	546897	5640789	1224	14:40	None	0	30	12	S3	Boulder	Gravel	Medium	No
Shulaps 1	2016-08-17	10	551022	5642014	806	12:45	None	0	26	11	S3	Cobble	Boulder	Medium	No
Shulaps 2	2016-08-17	10	545847	5638438	1432	15:20	None	0	30	12	S3	Boulder	Cobble	Low	Yes
Sidecar	2016-08-13	10	497434	5606939	1215	15:00	None	0	28	10	S6	Cobble	Gravel	Medium	Yes
Steep	2016-08-16	10	549292	5583770	1173	13:00	None	0	25	13	S3	Cobble	Gravel	Medium	No
Sucker	2016-08-14	10	511957	5634532	-	7:40	None	0	14	9	S3	Cobble	Gravel	Low	No
Truax 1	2016-08-15	10	521735	5633252	1513	8:45	None	0	9	6	S3	Boulder	Cobble	Low	Yes
Truax 2	2016-08-15	10	521810	5633670	1471	8:50	None	0	9	9	S5	Cobble	Gravel	Low	Yes
Truax 3	2016-08-15	10	521767	5632099	1423	8:40	None	0	9	6	S3	Cobble	Gravel	Medium	Yes
Van Horlick	2016-08-16	10	549561	5577902	1314	12:20	None	0	25	14	S3	Cobble	Gravel	Medium	Yes
Washout	2016-08-17	10	542303	5612586	492	14:50	None	0	30	14	S3	Boulder	Fines	Low	No
Waterfalls	2016-08-15	10	503914	5619249	1029	14:00	None	0	30	-	S3	Boulder	Gravel	High	No
White Saddle	2016-08-17	10	549602	5617437	255	14:00	None	0	28	13	S2	Gravel	Fines	High	No

[a]Not measured.
