# Supplementary material for: Expansion of the known distribution of the coastal tailed frog, Ascaphus truei, in British Columbia, Canada, using robust eDNA detection methods
Source: PLoS One. 2019 Mar 14;14(3):e0213849. doi: 10.1371/journal.pone.0213849 (PMC6417668; doi:10.1371/journal.pone.0213849)
Supplement: S2 Table — IntegritE-DNA tests were performed in four technical replicates and the eASTR4 test was done in eight technical replicates. Samples that failed the IntegritE-DNA test are indicated in orange. (PDF) [file pone.0213849.s002.pdf]

S2 Table. Individual sample results for the IntegritE-DNA and eASTR4 tests. IntegritE-DNA tests were performed in four technical replicates and the eASTR4 test was done in eight technical replicates. Samples that failed the IntegritE-DNA test are indicated in orange.

Location Name	Sample replicate	IntegritE-DNA test Frequency	IntegritE-DNA test Call	eASTR4 test Frequency	ASTR Call
Ainsworth's Folly	A	4/4	Pass	0/8	Negative
Ainsworth's Folly	B	4/4	Pass	2/8	Positive
Ault	A	4/4	Pass	1/8	Positive
Ault	B	4/4	Pass	0/8	Negative
Blowdown 1	A	4/4	Pass	1/8	Positive
Blowdown 1	B	4/4	Pass	0/8	Negative
Blowdown 2	A	4/4	Pass	0/8	Negative
Blowdown 2	B	4/4	Pass	0/8	Negative
Boulder	A	4/4	Pass	0/8	Negative
Boulder	B	4/4	Pass	0/8	Negative
Buck	A	4/4	Pass	6/8	Positive
Cadwallader 1	A	4/4	Pass	2/8	Positive
Cadwallader 1	B	4/4	Pass	0/8	Negative
Cadwallader 2	A	4/4	Pass	5/8	Positive
Cadwallader 3	A	4/4	Pass	0/8	Negative
Cadwallader 3	B	4/4	Pass	0/8	Negative
Carl	A	4/4	Pass	0/8	Negative
Carl	B	4/4	Pass	0/8	Negative
Casper	A	4/4	Pass	3/8	Positive
Cathy	A	4/4	Pass	1/8	Positive
Cathy	B	4/4	Pass	0/8	Negative
CatSki 1	A	4/4	Pass	1/8	Positive
CatSki 1	B	4/4	Pass	0/8	Negative
CatSki 2	A	4/4	Pass	6/8	Positive
CatSki 3	A	4/4	Pass	6/8	Positive
CatSki 4	A	4/4	Pass	5/8	Positive
CatSki 5	A	4/4	Pass	4/8	Positive
Cayoosh 1	A	4/4	Pass	5/8	Positive
Cayoosh 2	A	4/4	Pass	3/8	Positive
Channel	A	4/4	Pass	4/8	Positive
Cherise	A	4/4	Pass	2/8	Positive
Cherise	B	4/4	Pass	0/8	Negative
Chism	A	4/4	Pass	0/8	Negative
Chism	B	4/4	Pass	0/8	Negative
Conroy	A	4/4	Pass	0/8	Negative
Conroy	B	4/4	Pass	5/8	Positive
Copper	A	0/4	Fail	0/8	Negative
Copper	B	4/4	Pass	0/8	Negative
Crazy	A	4/4	Pass	0/8	Negative
Crazy	B	4/4	Pass	0/8	Negative
Doe	A	4/4	Pass	0/8	Negative
Doe	B	4/4	Pass	0/8	Negative
Downton 1	A	4/4	Pass	0/8	Negative
Downton 1	B	4/4	Pass	0/8	Negative
Downton 2	A	4/4	Pass	1/8	Positive
Downton 2	B	4/4	Pass	1/8	Positive
Fran's	A	4/4	Pass	3/8	Positive
Gott	A	4/4	Pass	4/8	Positive
Great Bear	A	4/4	Pass	5/8	Positive
Grey Rock	A	4/4	Pass	4/8	Positive

Gwyneth	A	4/4	Pass	2/8	Positive
Gwyneth	B	4/4	Pass	0/8	Negative
Hawthorne	A	4/4	Pass	1/8	Positive
Hawthorne	B	4/4	Pass	0/8	Negative
Haylemore 1	A	4/4	Pass	7/8	Positive
Haylemore 2	A	4/4	Pass	5/8	Positive
Holbrook 1	A	0/4	Fail	0/8	Negative
Holbrook 1	B	4/4	Pass	0/8	Negative
Holbrook 2	A	1/4	Fail	0/8	Negative
Holbrook 2	B	4/4	Pass	0/8	Negative
Hurley 1	A	4/4	Pass	8/8	Positive
Hurley 2	A	4/4	Pass	8/8	Positive
Hurley 3	A	4/4	Pass	2/8	Positive
Hurley 4	B	4/4	Pass	0/8	Negative
Hurley 4	A	4/4	Pass	7/8	Positive
Hurley 5	A	4/4	Pass	3/8	Positive
Ipoo	A	4/4	Pass	1/8	Positive
Ipoo	B	4/4	Pass	0/8	Negative
Joce	A	4/4	Pass	0/8	Negative
Joce	B	4/4	Pass	0/8	Negative
Kane	A	4/4	Pass	0/8	Negative
Kane	B	4/4	Pass	0/8	Negative
La Mare	A	4/4	Pass	1/8	Positive
La Mare	B	4/4	Pass	0/8	Negative
La Rochelle	A	4/4	Pass	8/8	Positive
Little	A	4/4	Pass	8/8	Positive
MacGillivray	A	4/4	Pass	1/8	Positive
MacGillivray	B	4/4	Pass	0/8	Negative
Marshall	A	4/4	Pass	0/8	Negative
Marshall	B	4/4	Pass	0/8	Negative
Mason	A	4/4	Pass	1/8	Positive
Mason	B	4/4	Pass	3/8	Positive
McParlon	A	4/4	Pass	1/8	Positive
McParlon	B	4/4	Pass	0/8	Negative
Ochre	A	4/4	Pass	1/8	Positive
Ochre	B	4/4	Pass	0/8	Negative
Pascall	A	4/4	Pass	1/8	Positive
Pascall	B	4/4	Pass	0/8	Negative
Paul	A	4/4	Pass	3/8	Positive
Pickup	A	4/4	Pass	3/8	Positive
Regehr	A	4/4	Pass	2/8	Positive
Regehr	B	4/4	Pass	4/8	Positive
Sebring	A	4/4	Pass	0/8	Negative
Sebring	B	4/4	Pass	0/8	Negative
Serpentine	A	4/4	Pass	2/8	Positive
Serpentine	B	4/4	Pass	0/8	Negative
Shulaps 1	A	4/4	Pass	7/8	Positive
Shulaps 2	A	4/4	Pass	0/8	Negative
Shulaps 2	B	4/4	Pass	0/8	Negative
Sidecar	A	4/4	Pass	8/8	Positive
Steep	A	4/4	Pass	5/8	Positive
Sucker	A	4/4	Pass	0/8	Negative
Sucker	B	4/4	Pass	0/8	Negative
Truax 1	A	4/4	Pass	3/8	Positive
Truax 2	A	4/4	Pass	1/8	Positive

Truax 2	B	4/4	Pass	1/8	Positive
Truax 3	A	4/4	Pass	1/8	Positive
Truax 3	B	4/4	Pass	0/8	Negative
Van Horlick	A	4/4	Pass	1/8	Positive
Van Horlick	B	4/4	Pass	0/8	Negative
Washout	A	4/4	Pass	0/8	Negative
Washout	B	4/4	Pass	3/8	Positive
Waterfalls	A	4/4	Pass	7/8	Positive
White Saddle	A	4/4	Pass	0/8	Negative
White Saddle	B	4/4	Pass	1/8	Positive
