# Supplementary material for: Expansion of the known distribution of the coastal tailed frog, Ascaphus truei, in British Columbia, Canada, using robust eDNA detection methods
Source: PLoS One. 2019 Mar 14;14(3):e0213849. doi: 10.1371/journal.pone.0213849 (PMC6417668; doi:10.1371/journal.pone.0213849)
Supplement: S3 Table — Historical data from four previous surveys within the region of interest (see Table 1 for details) were paired with eDNA sampling sites when the recorded location was within 2 km of the sampling site. (PDF) [file pone.0213849.s003.pdf]

S3 Table. Comparison of historical TCS results with eDNA results for coastal tailed frog. Historical data from four previous surveys within the region of interest (see Table 1 for details) were paired with eDNA sampling sites when the recorded location was within 2 km of the sampling site.

Location Name	Historical coastal tailed frog observed	eDNA result for site	Occurrence
Ainsworth's Folly	-[a]	Positive	New
Ault	No	Positive	New
Blowdown 1	-	Positive	New/Confirmed[b]
Blowdown 2	-	Negative	No
Boulder	-	Negative	No
Buck	No	Positive	New
Cadwallader 1	Yes	Positive	Confirmed
Cadwallader 2	No	Positive	New
Cadwallader 3	No	Negative	No
Carl	-	Negative	No
Casper	No	Positive	New
Cathy	-	Positive	New
CatSki 1	No	Positive	New
CatSki 2	-	Positive	New
CatSki 3	Yes	Positive	Confirmed
CatSki 4	Yes	Positive	Confirmed
CatSki 5	-	Positive	New
Cayoosh 1	Yes	Positive	Confirmed
Cayoosh 2	No	Positive	New
Channel	-	Positive	New/Confirmed[b]
Cherise	No	Positive	New
Chism	No	Negative	No
Conroy	-	Positive	New
Copper	-	Negative	No
Crazy	No	Negative	No
Doe	No	Negative	No
Downton 1	-	Negative	No
Downton 2	No	Positive	New
Fran's	-	Positive	New
Gott	-	Positive	New
Great Bear	-	Positive	New
Grey Rock	No	Positive	New
Gwyneth	-	Positive	New
Hawthorne	Yes	Positive	Confirmed
Haylemore 1	No	Positive	New
Haylemore 2	No	Positive	New
Holbrook 1	-	Negative	No
Holbrook 2	No	Negative	No
Hurley 1	No	Positive	New
Hurley 2	No	Positive	New
Hurley 3	No	Positive	New
Hurley 4	No	Positive	New
Hurley 5	No	Positive	New
Ipoo	No	Positive	New
Joce	-	Negative	No
Kane	-	Negative	No
La Mare	No	Positive	New
La Rochelle	-	Positive	New
Little	-	Positive	New
MacGillivray	No	Positive	New

Marshall	No	Negative	No
Mason	No	Positive	New
McParlon	-	Positive	New
Ochre	-	Positive	New
Pascall	-	Positive	New
Paul	No	Positive	New
Pickup	Yes	Positive	Confirmed
Regehr	Yes	Positive + Visual[c]	Confirmed
Sebring	No	Negative	No
Serpentine	No	Positive	New
Shulaps 1	No	Positive	New
Shulaps 2	-	Negative	No
Sidecar	Yes	Positive	Confirmed
Steep	-	Positive	New
Sucker	No	Negative	No
Truax 1	Yes	Positive	Confirmed
Truax 2	No	Positive	New
Truax 3	No	Positive	New
Van Horlick	No	Positive	New
Washout	No	Positive	New
Waterfalls	No	Positive	New
White Saddle	-	Positive	New

[a]Not examined within the four historical TCS studies.

[b]Observed outside of the four historical TCS studies (P. Friele and F. Iredale, personal communication).

[c]While sampling for eDNA.
